# Persistent hepatitis burden in a geographically isolated and medically underserved population: a community-based study

**DOI:** 10.1038/s41598-026-48166-2

**Published:** 2026-04-24

**Authors:** Dong-Hee Ryu, Eun-Young Lee, Soo-Jung Ha, Nam-soo Hong

**Affiliations:** 1 Department of Preventive Medicine, Daegu Catholic University School of Medicine, Daegu, 42472 Republic of Korea; 2 Daegu-Gyeongbuk Regional Cancer Center, Kyungpook National University Medical Center, Daegu, 41404 Republic of Korea; 3 Ulleung Public Community Health Center, Gyeongbuk, 40217 Republic of Korea; 4 Department of Preventive Medicine, School of Medicine, Kyungpook National University, 680 Gukchaebosang-ro, Jung-gu, Daegu, 41944 Republic of Korea

**Keywords:** Early detection of cancer, Health behavior, Health risk behaviors, Hepatitis B, Hepatitis C, Liver neoplasm, Cancer, Diseases, Gastroenterology, Health care, Risk factors

## Abstract

Ulleung County is a geographically isolated and medically underserved region in South Korea with a persistently high incidence of liver cancer. Using a community-based approach, this study aimed to estimate the prevalence of hepatitis B virus (HBV) and hepatitis C virus (HCV) infections and to identify associated risk factors. As part of the Ulleung Liver Cancer Prevention and Management Project, an interagency workgroup implemented community-based screening and health behavior surveys. Between 2019 and 2022, a total of 1,792 residents aged ≥ 40 years voluntarily participated, and data from 1,684 participants were analyzed. The prevalence of hepatitis B surface antigen (HBsAg) and hepatitis C virus antibody (anti-HCV) positivity was 7.19% and 1.37%, respectively. Lower educational attainment and lifetime smoking were significantly associated with HBsAg positivity, whereas cupping therapy performed at non-medical institutions was associated with anti-HCV positivity. HBsAg positivity was lower among individuals with tattoos. Although the high prevalence of HBV infection alone may not fully explain the elevated incidence of liver cancer in Ulleung County, it likely represents an important contributing factor. These findings highlight the value of community-based screening strategies for informing tailored hepatitis prevention efforts in geographically isolated populations.

**Supplementary Information:**

The online version contains supplementary material available at 10.1038/s41598-026-48166-2.

## Introduction

As of 2020, the age-standardized liver cancer mortality rate in South Korea was 9.9 per 100,000 people which was the highest in the world^1^. According to the World Health Organization (WHO), viral hepatitis causes more than one million deaths per year worldwide, including deaths due to liver cancer caused by hepatitis infection^2^. As such, hepatitis viruses are well-known risk factors for liver cancer. Chronic hepatitis virus infections are associated with hepatic fibrosis and the eventual development of hepato-cellular carcinoma^3,4^. The WHO estimates that in 2022, 254 million people were living with chronic hepatitis B infection, with 1.2 million new cases occurring annually^2^. Moreover, it is estimated that 50 million people are living with chronic hepatitis C virus infections globally, with around 1.0 million new infections each year^5^. Approximately 1.1 million deaths and 242,000 deaths occurred due to hepatitis B and hepatitis C, respectively, primarily due to liver cirrhosis and hepatocellular carcinoma^5^.

**Fig. 1:**
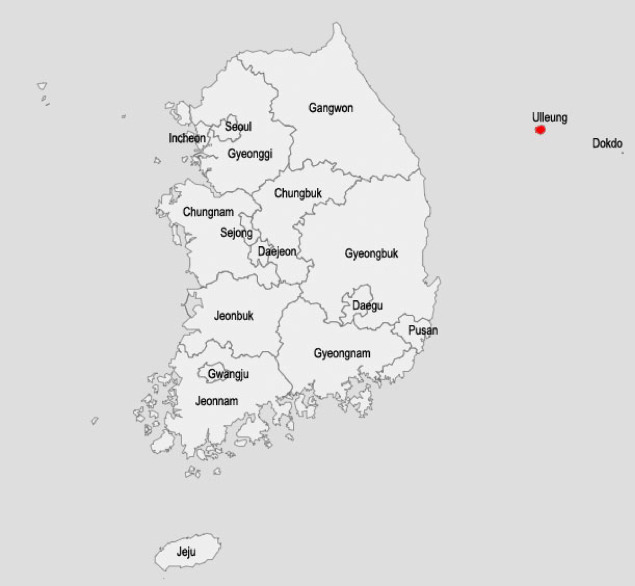
Map of South Korea, with Ulleung shaded in red.

Ulleung County is one of the most medically vulnerable areas in South Korea. This geographically isolated island, located in Gyeongbuk province (Fig. 1), had a population of 8,996 residents (4,976 males and 4,020 females) across 5,479 households as of 2022^6^. Healthcare accessibility in the county is severely limited, with only three private medical institutions, two oriental medicine clinics, and one dental clinic serving the entire population. The Ulleung Public Community Health Center functions as the primary healthcare provider, delivering both general medical care and public health services. Despite its small population, Ulleung County has consistently reported a disproportionately high incidence of liver cancer. The age-standardized liver cancer incidence was 102.5 per 100,000 population during 1999–2003 and remained elevated at 61.5 during 2014–2018, while the national figures for the same period were 50.1 and 35.1, respectively^7^. However, population-based data on the prevalence of viral hepatitis in this region remain limited.

In response to the persistently high burden of liver cancer, the Daegu-Gyeongbuk Regional Cancer Center initiated the Ulleung Liver Cancer Prevention and Management Project in 2018. This multi-institutional initiative was implemented through an interagency workgroup, which coordinated prevention education, health behavior interventions, and collaboration among local public health authorities and policy stakeholders. Findings from the early phase of the project highlighted the need for systematic epidemiological data on hepatitis B and C virus infections within the community^8^.

As part of the Ulleung Liver Cancer Prevention and Management Project, this study aimed to estimate the prevalence of community hepatitis B and C viruses and to identify the associated risk factors. Because liver cancer screening in Korea is recommended starting at age 40 for high-risk populations, the present study focused on residents aged ≥ 40 years.

## Methods

### Subject recruitment

To enhance community-wide participation and minimize selection bias, recruitment was conducted through multiple outreach strategies coordinated by an interagency workgroup. These included public health announcements, visual media at Ulleung Port, banners across the island, and informational leaflets distributed at the Ulleung Public Community Health Center. Residents aged 40 years and older who became aware of the program through these announcements were invited to voluntarily visit the Ulleung Public Community Health Center to participate in hepatitis screening and the health behavior survey.

As of 2019, the number of registered residents in Ulleung was 9,617 (5,230 males and 4,387 females), and people aged 40 years and older accounted for 70.2% (6,750) of this population^6^. Between 2019 and 2022, a total of 1,792 residents aged 40 years or older voluntarily participated in the study, representing approximately 27% of the eligible population. The age distribution of study participants differed from that of the underlying Ulleung County population aged ≥ 40 years, with relatively greater participation among individuals aged 40–69 years and lower participation among those aged ≥ 70 years (Supplementary Table S1).

Trained nurses explained the study purpose and procedures, and written informed consent was obtained from all participants. All methods were performed in accordance with the relevant guidelines and regulations. The study protocol was approved by the Institutional Review Board of Kyungpook National University Medical Center (approval no. knuh2018-11-005; November 21, 2019).

### Serologic tests

Approximately 5 mL of venous blood was drawn from all participants by a trained nurse. All the samples were subjected to hepatitis B surface antigen (HBsAg) and hepatitis C virus antibody (anti-HCV) tests. HBsAg and anti-HCV screening were performed using the Cobas e801 module (Roche Diagnostic International Ltd., Rotkreuz, Switzerland), and Alinity (Abbott Diagnostics, Wiesbaden, Germany), respectively. According to the manufacturer’s specifications, the assays used in this study have high sensitivity and specificity for detecting HBsAg and anti-HCV antibodies. All laboratory analyses were conducted at a certified external laboratory (Seoul Clinical Laboratories) following established quality control protocols.

### Health behavior survey

The health behavior survey was administered using a previously established questionnaire^8^. The questionnaire was reviewed and approved by members of the Interagency Workgroup and an epidemiologist^8^. The items were categorized into general characteristics, general health behaviors, and risky health behaviors.

The general characteristics included sex, age (40–49, 50–59, 60–69, and ≥ 70 years), level of education (≤ elementary, junior high school, high school, and ≥ college), average monthly household income (< 500, 500–999, 1000–1999, 2000–2999, 3000–3999, and ≥ 4000), and duration of residence in Ulleung (< 1 year, 1–9 years, and ≥ 10 years).

The general health behaviors included cigarette smoking (never, former, or current), alcohol consumption (no, moderate, or heavy), obesity (normal, overweight, or obese), and regular cancer screening. Heavy alcohol consumption was defined as seven or more drinks for men and five or more drinks for women at least twice per week^9^. Moderate drinkers were defined as participants who were neither non-drinkers nor heavy drinkers^9^. Obesity status was based on body mass index (BMI); height was measured using the BSM330 instrument (Biospace Inc., Seoul, South Korea); and body composition was measured using the InBody770 analyzer (Biospace Inc., Seoul, South Korea). BMI was calculated as weight divided by height squared (kg/m^2^) and categorized according to WHO criteria for Asian populations as normal (< 23.0 kg/m^2^), overweight (23.0–24.9 kg/m^2^) and obese (≥ 25 kg/m^2^).

Risky health behaviors included tattooing, piercing, acupuncture, cupping therapy, transfusion, dialysis, doctor-diagnosed diabetes mellitus, and lack of knowledge of liver diseases (hepatitis B, hepatitis C, and liver cirrhosis)^8,10^. Information on injection drug use was collected in the survey; however, only nine participants reported such history, which precluded meaningful statistical analysis. Regarding the items on knowledge of liver diseases, detailed information could not be obtained owing to methodological limitations; therefore, each participant was simply asked whether they knew about the diseases; the response of “yes” to the questions indicated the participant had the necessary knowledge. Acupuncture and cupping therapy can cause blood-borne infections^11–13^; hence, these therapies should be performed only at certified medical institutions, but they are sometimes illegally performed at non-medical institutions in Korea^14^. Therefore, the participants were asked whether they received acupuncture or cupping therapy at uncertified places.

### Statistical analysis

The participants with missing values for the main variables were excluded from the analysis. To assess the impact of missing data, we compared the demographic characteristics of the included and excluded participants. This comparison showed no significant differences, suggesting that the missing data may not have caused substantial bias. Age-standardized prevalence was calculated using the age distribution of the Ulleung County population obtained from the Korean Statistical Information Service (KOSIS). We used the chi-square test or Fisher’s exact test for frequency analysis. Multivariable logistic regression analyses were performed to examine the association between HBsAg or anti-HCV positivity and health behavioral characteristics. Additional analyses were performed after adjusting for sex, age, education, monthly household income, and residential period. All statistical analyses were performed using SAS version 9.4 (SAS Institute Inc., Cary, NC, USA), and statistical significance was set at *p* < 0.05.

## Results

### Participant characteristics

A total of 1,684 individuals were included in the final analysis (Table 1); 67% were enrolled in the study before the coronavirus disease (COVID-19) pandemic in 2019. The participation rate decreased after 2020 owing to the COVID-19 pandemic. However, no statistically significant differences were observed in sex or age distribution between participants enrolled before and during the pandemic (sex: *p* = 0.184; age: *p* = 0.142; data not shown).

**Table 1:** General characteristics.

Variables	*n* (%)
Total	1684 (100.0)
Sex	
Male	840 (49.9)
Female	844 (50.1)
Age	
40–49	417 (24.8)
50–59	531 (31.5)
60–69	726 (43.1)
≥70	10 (0.6)
Education	
≤Elementary	218 (13.0)
Junior high	331 (19.7)
High	660 (39.2)
≥College	475 (28.2)
Monthly household income (1000 KRW)	
<500	256 (15.2)
500–999	170 (10.1)
1000–1999	373 (22.2)
2000–2999	330 (19.6)
3000–3999	227 (13.5)
≥4000	328 (19.5)
Residential period in Ulleung	
<1 year	75 (4.5)
1–9 years	412 (24.5)
≥10 years	1197 (71.1)

### HBsAg and anti-HCV prevalence

Most participants did not report a prior diagnosis of liver cirrhosis or liver cancer. Of 1,684 individuals, 118 reported being hepatitis B virus (HBV) carriers, and 36 reported receiving regular treatment for liver disease. In addition, 55.3% reported having received HBV vaccination (Table 2).

**Table 2:** Reported liver conditions.

Variables	*n* (%)
Total	1684 (100.0)
Diagnosed with liver cancer	4 (0.2)
Diagnosed with liver cirrhosis	5 (0.3)
Diagnosed with chronic hepatitis C	9 (0.5)
Diagnosed with chronic hepatitis B	22 (1.3)
Diagnosed as HBV carriers	118 (7.0)
Undergoing treatment for liver disease	36 (2.1)
Reported HBV vaccination	
No	718 (42.6)
Yes	932 (55.3)
No antibody generation despite vaccination	34 (2.0)

### Factors associated with HBsAg and anti-HCV positivity

Serological testing identified 121 participants as HBsAg-positive (7.19%, 95% CI: 6.0–8.5) and 23 as anti-HCV-positive (1.37%, 95% CI: 0.9–2.0). HBsAg positivity was more frequently observed among men, participants aged ≥ 60 years, those with lower educational attainment, lower household income, and longer residence in the county. In contrast, anti-HCV positivity did not show a statistically significant association with any of the general characteristics analyzed (Table 3).

**Table 3:** Seropositivity for HBsAg and anti-HCV according to general characteristics.

Variables	HBsAg positivity			Anti-HCV positivity		
	*n* (%)	Crude OR (95% CI)	Adjusted OR (95% CI)	*n* (%)	Crude OR (95% CI)	Adjusted OR (95% CI)
Total	121 (7.19)	-		23 (1.37)	-	
Sex						
Male	66 (7.86)	Reference	-	10 (1.19)	Reference	-
Female	55 (6.52)	0.83 (0.59, 1.18)	-	13 (1.54)	1.41 (0.62, 3.19)	-
Age						
40–49	22 (5.45)	Reference	-	5 (1.24)	Reference	-
50–59	33 (6.21)	1.15 (0.69, 1.92)	-	8 (1.51)	1.27 (0.41, 3.91)	-
60–69	62 (8.54)	1.50 (0.95, 2.38)	-	9 (1.24)	1.16 (0.39, 3.41)	-
≥ 70	4 (40.00)	13.18 (4.14, 41.94)	-	1 (10.00)	7.28 (0.79, 67.22)	-
Education						
≤Elementary	27 (12.39)	3.31 (1.86, 5.87)	3.58 (1.79–7.15)	5 (2.29)	1.59 (0.50, 5.05)	1.07 (0.25–4.53)
Jr. high	29 (8.76)	2.34 (1.34, 4.08)	2.55 (1.35–4.81)	3 (0.91)	0.61 (0.16, 2.37)	0.46 (0.10–2.11)
High	43 (6.52)	1.79 (1.08, 2.99)	1.90 (1.12–3.21)	8 (1.21)	0.91 (0.34, 2.45)	0.78 (0.28–2.19)
≥College	22 (4.63)	Reference	Reference	7 (1.47)	Reference	Reference
Monthly household income (1000 KRW)						
< 500	22 (8.59)	1.89 (1.01, 3.53)	1.71 (0.85–3.43)	1 (0.39)	0.43 (0.04, 4.16)	0.34 (0.03–3.55)
500–999	19 (11.18)	2.24 (1.16, 4.36)	2.06 (1.01–4.21)	1 (0.59)	1.27 (0.21, 7.65)	1.04 (0.16–6.86)
1000–1999	28 (7.51)	1.65 (0.91, 2.99)	1.51 (0.81–2.82)	10 (2.68)	2.95 (0.81, 10.82)	2.47 (0.63–9.62)
2000–2999	20 (6.06)	1.43 (0.77, 2.65)	1.41 (0.75–2.64)	7 (2.12)	2.28 (0.59, 8.89)	2.22 (0.56–8.74)
3000–3999	15 (6.61)	1.44 (0.73, 2.83)	1.43 (0.73–2.81)	1 (0.44)	0.47 (0.05, 4.52)	0.47 (0.05–4.57)
≥ 4000	17 (5.18)	Reference	Reference	3 (0.91)	Reference	Reference
Residential period in Ulleung						
< 1 year	4 (5.33)	0.54 (0.19, 1.49)	0.57 (0.20–1.60)	2 (2.67)	2.37 (0.53–10.61)	3.10 (0.66–14.52)
1–9 years	14 (3.40)	0.45 (0.27, 0.73)	0.48 (0.29–0.80)	8 (1.94)	1.67 (0.69–4.00)	2.00 (0.80–5.01)
≥ 10 years	103 (8.60)	Reference	Reference	13 (1.09)	Reference	Reference

After adjustment for sex, age, education, household income, and residential period, former and current smokers had significantly higher odds of HBsAg positivity compared with non-smokers (adjusted odds ratio [aOR] = 2.06, 95% CI:1.12–3.80 and aOR = 1.86, 95% CI:1.03–3.35). Alcohol consumption, obesity, and regular cancer screening were not significantly associated with HBsAg or anti-HCV positivity (Table 4).

**Table 4:** General health behaviors according to seropositivity of HBsAg and Anti-HCV.

Variables	HBsAg positivity			Anti-HCV positivity		
	*n* (%)	Crude OR (95% CI)	Adjusted OR (95% CI)	*n* (%)	Crude OR (95% CI)	Adjusted OR (95% CI)
Cigarette smoking						
Never	57 (5.83)	Reference	Reference	14 (1.43)	Reference	Reference
Former	35 (10.23)	1.69 (1.11, 2.57)	2.06 (1.12, 3.80)	1 (0.29)	0.19 (0.03, 1.46)	1.70 (0.52, 5.56)
Current	29 (7.97)	1.44 (0.95, 2.20)	1.86 (1.03, 3.35)	8 (2.20)	1.40 (0.59, 3.33)	0.28 (0.03, 2.53)
Alcohol consumption						
No	54 (9.08)	Reference	Reference	11 (1.85)	Reference	Reference
Moderate	42 (5.48)	0.61 (0.42, 0.91)	0.70 (0.47, 1.06)	9 (1.17)	0.70 (0.30, 1.67)	0.74 (0.30, 1.85)
Heavy	25 (7.74)	0.78 (0.49, 1.26)	0.83 (0.48, 1.41)	3 (0.93)	0.49 (0.14, 1.76)	0.59 (0.14, 2.43)
Obesity						
Normal	39 (7.29)	Reference	Reference	7 (1.31)	Reference	Reference
Overweight	31 (7.47)	0.85 (0.53, 1.35)	0.81 (0.50, 1.31)	4 (0.96)	0.66 (0.20, 2.21)	0.69 (0.20, 2.38)
Obese	51(6.95)	0.94 (0.64, 1.40)	0.90 (0.60, 1.35)	12 (1.63)	1.12 (0.46, 2.76)	1.31 (0.51, 3.34)
Cancer screening						
No	18 (4.93)	0.61 (0.38, 0.99)	0.63 (0.38, 1.04)	9 (2.47)	2.17 (0.94, 4.99)	2.44 (0.99, 6.04)
Yes	103 (7.81)	Reference	Reference	14 (1.06)	Reference	Reference

Regarding risky health behaviors, tattooing and knowledge of liver diseases were significantly associated with HBsAg positivity. In contrast, anti-HCV positivity was significantly associated with a history of cupping therapy performed at non-medical institutions (Table 5).

**Table 5:** Risky health behaviors according to seropositivity of HBsAg and Anti-HCV.

Variables	HBsAg positivity			Anti-HCV positivity		
	*n* (%)	Crude OR (95% CI)	Adjusted OR (95% CI)	*n* (%)	Crude OR (95% CI)	Adjusted OR (95% CI)
Tattooing						
No	86 (8.29)	Reference	Reference	13 (1.25)	Reference	Reference
Yes	35 (5.41)	0.62 (0.42, 0.90)	0.53 (0.33, 0.85)	10 (1.55)	1.36 (0.61, 3.06)	1.12 (0.41, 3.13)
Piercing						
No	86 (7.78)	Reference	Reference	12 (1.08)	Reference	Reference
Yes	35 (6.06)	0.73 (0.50, 1.07)	0.83 (0.50, 1.37)	11 (1.90)	1.65 (0.73, 3.70)	1.83 (0.56, 5.91)
Acupuncture at certified clinics						
No	24 (7.12)	Reference	Reference	2 (0.59)	Reference	Reference
Yes	97 (7.20)	0.96 (0.62, 1.46)	0.94 (0.61, 1.45)	21 (1.56)	2.78 (0.65, 11.88)	2.90 (0.67, 12.56)
Acupuncture at uncertified institutions						
No	118 (7.34)	Reference	Reference	21 (1.31)	Reference	Reference
Yes	3 (3.90)	0.43 (0.13, 1.38)	0.47 (0.15, 1.51)	2 (2.60)	1.89 (0.44, 8.19)	1.91 (0.43, 8.46)
Cupping therapy at certified clinics						
No	59 (7.69)	Reference	Reference	8 (1.04)	Reference	Reference
Yes	62 (6.76)	0.92 (0.65, 1.30)	0.90 (0.63, 1.28)	15 (1.64)	1.40 (0.61, 3.23)	1.37 (0.59, 3.19)
Cupping therapy at uncertified institutions						
No	119 (7.36)	Reference	Reference	19 (1.18)	Reference	Reference
Yes	2 (2.99)	0.32 (0.08, 1.33)	0.36 (0.09, 1.49)	4 (5.97)	4.92 (1.64, 14.79)	4.91 (1.55, 15.55)
Transfusion						
No	103 (7.15)	Reference	Reference	18 (1.25)	Reference	Reference
Yes	17 (7.69)	1.21 (0.75, 1.95)	1.30 (0.80, 2.11)	5 (2.26)	1.65 (0.61, 4.45)	1.68 (0.61, 4.68)
Unknown	1 (4.55)	0.55 (0.07, 4.14)	0.49 (0.07, 3.73)	-	-	-
Dialysis						
No	119 (7.10)	Reference	Reference	23 (1.37)	Reference	Reference
Yes	2 (25.00)	3.40 (0.70, 16.50)	3.22 (0.64, 16.19)	-	-	-
Doctor-diagnosed diabetes mellitus						
No	99 (6.89)	Reference	Reference	18 (1.25)	Reference	Reference
Yes	22 (8.91)	1.18 (0.74, 1.89)	0.95 (0.58, 1.55)	5 (2.02)	1.56 (0.58, 4.22)	1.57 (0.55, 4.48)
Understanding hepatitis B						
No	27 (3.65)	Reference	Reference	10 (1.35)	Reference	Reference
Yes	94 (9.96)	2.95 (1.95, 4.44)	4.12 (2.66, 6.40)	13 (1.38)	1.09 (0.48, 2.47)	1.02 (0.44, 2.40)
Understanding hepatitis C						
No	73 (6.25)	Reference	Reference	13 (1.11)	Reference	Reference
Yes	48 (9.30)	1.44 (1.01, 2.06)	1.79 (1.22, 2.62)	10 (1.94)	1.90 (0.85, 4.27)	2.01 (0.87, 4.69)
Understanding liver cirrhosis						
No	33 (7.47)	Reference	Reference	10 (2.26)	Reference	Reference
Yes	88 (7.09)	1.07 (0.72, 1.60)	1.43 (0.93, 2.19)	13 (1.05)	0.49 (0.22, 1.12)	0.46 (0.19, 1.09)

## Discussion

In this community-based study conducted in a medically vulnerable and geographically isolated region, we observed a markedly high prevalence of hepatitis B virus infection, with an HBsAg positivity rate of 7.19%. This level is substantially higher than recent national estimates and comparable to those reported in South Korea during the 1980 s, prior to the implementation of widespread hepatitis B vaccination programs^15–17^. Following the introduction of a national vaccination program in 1983 and universal immunization in 1998, HBsAg positivity in the general Korean population declined to approximately 2.9% in the 2010s^18^. Similarly, the estimated HBsAg prevalence in East Asia in 2019 was 3.02%^19^, which is less than half of the prevalence observed in the present study. Age-specific estimates from the 2021 Korea National Health and Nutrition Examination Survey further indicated that HBsAg positivity in the general population remains below 6% across adult age groups (40–49 years: 3.9%, 50–59 years: 5.7%, 60–69 years: 2.4%, ≥ 70 years: 3.0%)^20^, reinforcing the disproportionately high burden observed in Ulleung County. Together, these findings suggest that the benefits of national hepatitis B prevention strategies may not yet have been fully realized in this geographically isolated community. To confirm the prevalence of HBsAg in the community, additional research targeting all age groups, including children, adolescents, or young people is necessary. From a public health perspective, these findings highlight the potential value of targeted hepatitis screening strategies in geographically isolated communities. Universal screening may not always be feasible in resource-limited settings such as Ulleung County. The identification of population characteristics associated with HBV positivity may help guide more focused screening and prevention efforts in medically underserved regions.

In contrast to the markedly high prevalence of hepatitis B virus infection, the prevalence of hepatitis C virus infection in this study was relatively modest. The anti-HCV positivity rate was 1.37%, similar to that reported in previous population-based studies in Korea^21–23^. By comparison, a community-based study conducted in Jeonnam Province, a region with a particularly high incidence of liver cancer, reported an anti-HCV positivity rate as high as 5.5%^23^. Previous studies have suggested that regions with a high prevalence of hepatitis C virus may experience a higher burden of liver cancer^23^. Nationally, the prevalence of anti-HCV positivity in South Korea has been estimated at 0.6–0.8% and has been shown to increase with age in both sexes^24^. In this context, the relatively higher anti-HCV positivity observed in the present study may be partially explained by the older age distribution of the study population, with approximately half of participants aged 60 years or older. Nevertheless, unlike hepatitis B virus infection, an association between anti-HCV positivity and the high incidence of liver cancer in Ulleung County was not evident.

Beyond regional differences in viral hepatitis prevalence, sociodemographic factors also appeared to play an important role in shaping hepatitis B virus infection risk in this population. In the present study, HBsAg prevalence increased significantly as the educational level decreased, a finding consistent with previous research from Taiwan reporting inverse associations between educational attainment and both HBsAg and anti-HCV positivity^25^. However, low educational level was identified as a risk factor for hepatitis B virus infection but not for hepatitis C virus infection in the present study. This pattern may be explained by differences in prevention mechanisms, as effective vaccines are available for hepatitis B but not for hepatitis C. Higher educational attainment may therefore facilitate access to vaccination and preventive health information related to hepatitis B. Additionally, educational level may be correlated with knowledge of hepatitis B, whereas understanding of hepatitis C remains limited in the general population. This interpretation is supported by the present findings, which demonstrated an association between knowledge of liver diseases and HBsAg positivity even after adjustment for sociodemographic characteristics, while no such association was observed for anti-HCV positivity. This finding highlights the importance of improving public awareness and health literacy regarding viral hepatitis, which may facilitate earlier screening and preventive health behaviors in underserved communities.

Behavioral factors may be associated with differences in chronic viral hepatitis prevalence and its progression to liver disease. In this study, 41.9% of participants reported lifetime smoking, a proportion comparable to that reported in the 2022 Ulleung Community Health Survey (39.5%)^26^. Previous studies have identified both chronic viral hepatitis and cigarette smoking as risk factors for hepatocellular carcinoma^27–29^, and a meta-analysis published in 2010 reported synergistic effects of HBV or HCV infection and smoking on hepatocellular carcinoma risk^29^. The significant association observed between HBsAg positivity and lifetime smoking identified in this study supports the results of the previous studies. In contrast, no significant association was found between anti-HCV positivity and smoking status. Although this result differs from a previous study reporting smoking as a risk factor among HCV-seropositive individuals but not among those with HBV infection^25^, the findings collectively indicate that the relationship between smoking and chronic viral hepatitis may vary by viral type and population context, requiring further investigation.

Not all behaviors traditionally considered to be high-risk for blood-borne infections were associated with viral hepatitis in this study. Unexpectedly, the odds of HBsAg positivity were significantly lower among individuals with tattoos, and no significant association was observed between tattooing and anti-HCV positivity. These findings are consistent with previous studies suggesting that tattooing itself is not independently associated with chronic viral hepatitis^30^. Since HBsAg reflects chronic HBV infection rather than cumulative exposure, it may not be an optimal marker for evaluating infection risk related to adult behaviors such as tattooing. Future studies incorporating anti-HBc testing would help better assess the relationship between behavioral exposures and HBV infection. Accordingly, caution should be exercised when using tattooing as a surrogate for other risky health behaviors, such as piercing or traditional medical practices (acupuncture or cupping therapy) performed outside regulated medical settings.

However, certain traditional medical practices conducted at non-medical institutions remained relevant risk factors, particularly for hepatitis C virus infection. Cupping therapy performed at non-medical institutions was significantly associated with anti-HCV positivity, suggesting that inadequate infection control practices may contribute to viral transmission. Viral transmission through percutaneous exposure is a well-established route for hepatitis C virus infection, whereas hepatitis B virus transmission occurs commonly through perinatal or sexual routes^31^. The absence of an association between cupping therapy and HBsAg positivity, as well as the lack of association between acupuncture at non-medical institutions and anti-HCV positivity, may reflect differences in procedural sterility. These findings highlight the need for further in-depth research on infection control practices in traditional medical procedures and their potential role in viral hepatitis transmission.

Several limitations should be considered when interpreting the findings of this study. First, the analysis relied on community-based screening and survey data without comparison to a control region, limiting causal inference. Second, participation in the screening program was voluntary and recruitment relied on community outreach rather than random sampling; therefore selection bias cannot be completely excluded. Individuals with greater health concerns may have been more likely to participate, which could have resulted in an overestimation of hepatitis prevalence. Since the age distribution of participants did not fully match that of the source population, particularly for adults aged ≥ 70 years, prevalence estimates should be interpreted with caution. Third, standardized questionnaires could not be used, and self-developed survey instruments were administered in a self-report format, raising the possibility of response bias. Finally, this study was designed to provide a broad overview of hepatitis B and C prevalence and associated factors rather than an in-depth etiological analysis. Nevertheless, to our knowledge, this is the first study to examine chronic viral hepatitis and its associated factors in Ulleung County. Moreover, as part of a multi-institutional project, this study demonstrates a feasible research framework for investigating infectious diseases in medically underserved and geographically isolated communities.

Taken together, the findings of this study indicate that the epidemiology and risk factors of hepatitis B and C virus infections differ substantially in Ulleung County. While a high HBsAg positivity rate alone may not fully explain the region’s elevated liver cancer incidence, it likely represents an important contributing factor. In contrast, the relatively low prevalence of hepatitis C virus infection does not appear to account for the high liver cancer burden observed in this community. These findings may help inform targeted screening strategies and health education interventions for populations living in medically underserved and geographically isolated regions. Early detection of viral hepatitis not only enables timely anti-viral treatment but may also reduce progression to liver cirrhosis and hepatocellular carcinoma.

## Supplementary Information

Below is the link to the electronic supplementary material.

Supplementary Material 1

## Data Availability

Data were generated by the authors and available from the corresponding author upon reasonable requests.
