# Supplementary material for: Persistent hepatitis burden in a geographically isolated and medically underserved population: a community-based study
Source: Sci Rep. 2026 Apr 24;16:19878. doi: 10.1038/s41598-026-48166-2 (PMC13316040; doi:10.1038/s41598-026-48166-2)
Supplement: Supplementary file 1 — Supplementary Material 1 [file 41598_2026_48166_MOESM1_ESM.docx]

| **Supplementary Table S1.** Comparison of age distribution between study participants and the underlying Ulleung County population aged ≥40 years |
| --- |

| **Age group (years)** | **Ullueng County population, n(%)** | **Study participants, n(%)** |
| --- | --- | --- |
| 40-49 | 1,225 (18.5) | 417 (24.8) |
| 50-59 | 1,794 (27.1) | 531 (31.5) |
| 60-69 | 1,912 (28.9) | 726 (43.1) |
| ≥70 | 1,677 (25.4) | 10 (0.6) |
| Total | 6,608 (100.0) | 1,684 (100.0) |

Population data were obtained from the Korean Statistical Information Service (KOSIS), UIleung County resident registration population (2022). Study participant distribution was based on the final analytic sample.
